# A Rare Case of Untreated Wilson’s Disease in a Teen With Lethal Exit: Morphological Findings From an Autopsy Study

**DOI:** 10.7759/cureus.68838

**Published:** 2024-09-06

**Authors:** Anastasia I Bekyarova, Ina Kobakova, Snejana Spasova

**Affiliations:** 1 Department of General and Clinical Pathology, Forensic Medicine and Deontology, Medical University of Varna, Varna, BGR; 2 Department of General and Clinical Pathology, Forensic Medicine and Deontology, Dr. Marko Markov Specialized Hospital for Treatment of Oncological Diseases, Varna, BGR

**Keywords:** autopsy, minority background, morphology, untreated wilson's disease, wilson's disease in children

## Abstract

Wilson's disease (WD) is an autosomal recessive genetic disorder caused by more than 50 different mutations in the APT7B gene. A defect in the gene product results in copper accumulation mainly in the liver, basal ganglia in the brain, cornea, kidneys, and heart, leading to dysfunction and eventually organ failure. We present a case of a 15-year-old male with a minority background who did not receive any form of treatment and ultimately succumbed to the disease. He was previously hospitalized due to suspected autoimmune-mediated acute liver failure (ALF) with positive antinuclear autoantibodies. Abdominal ultrasound revealed uneven contours and diffusely abnormal structure of the liver, interpreted as liver cirrhosis (LC), and splenomegaly. In view of WD as a potential differential diagnosis, a genetic consultation recommended the performance of genetic testing. The patient received symptomatic and corticosteroid therapy and was discharged from the hospital with improved general status. Three days later, the teen experienced deterioration and was readmitted to the hospital in a critical state. Reanimation measures had a temporary effect and ultimately exitus letalis was registered. The autopsy study revealed mixed micronodular and macronodular LC, chronic steatohepatitis, hepatosplenomegaly, ascites, icterus, gynecomastia, telangiectasias, subcutaneous hemorrhages, absence of male pattern body hair, hypogonadism, and chronic calculous cholecystitis as a result of untreated WD. Complications of the main disease appeared to be hepatorenal syndrome, severe bilateral purulent-hemorrhagic pneumonia probably with mixed etiology, acute cardiac failure with congestive changes in all internal organs, pleural and pericardial effusions, pulmonary edema, and cerebral edema with tonsillar herniation. The ATP7B gene sequencing supported the clinical diagnosis and the autopsy suspicion of WD, showing that the boy was homozygous for an H1069Q mutation.

## Introduction

Wilson's disease (WD), also known as hepatolenticular degeneration, is an autosomal recessive genetic disorder caused by more than 50 different mutations in the APT7B gene [1]. The gene encodes the transmembrane copper-transporting ATPase, an enzyme responsible for the incorporation of copper into ceruloplasmin and its excretion into the bile. A defect in the gene product results in copper accumulation mainly in the liver, basal ganglia in the brain, cornea, kidneys, and heart, leading to dysfunction and eventually organ failure [2]. Patients usually become symptomatic between the ages of 5 and 35 years and present with progressive liver disease and neurological and psychiatric symptoms. The presentation appears to be age- and sex-dependent, as liver cirrhosis (LC) is most commonly observed in adults and females having a greater risk of acute liver failure (ALF) [3]. Diagnosis is based primarily on clinical and laboratory parameters and genetic testing. Despite its genetic nature, WD is potentially treatable with chelators, plasmapheresis in ALF, liver transplantation for patients with advanced LC, and gene therapy as a future therapeutic strategy. Avoiding copper-rich foods (e.g., seafood, liver, nuts, mushrooms, and chocolate) might also be beneficial. Compliance with therapy is essential to achieve satisfactory results and an optimistic prognosis [4]. This report aims to present the morphological changes in a case study of WD in a 15-year-old child with a minority background who did not receive any form of treatment and ultimately succumbed to the disease.

## Case presentation

We present a case of a 15-year-old male who was previously hospitalized due to suspected autoimmune-mediated ALF with positive antinuclear autoantibodies. He was admitted to the hospital with a two-day history of pitting edema of the lower limbs. On examination, the patient exhibited hypersthenic body habitus, icterus, noticeable on the scleras, with no data for a Kayser-Fleischer ring in the corneas. Complete blood count tests showed signs of anemia, thrombocytopenia, low total protein, mildly elevated alanine transaminase (ALT) and aspartate aminotransferase (AST) levels (AST/ALT ratio was <1.0), high total bilirubin levels (280 umol/L), reduction in cholinesterase activity, and low ceruloplasmin. Urine analysis indicated slightly elevated urobilinogen levels. Abdominal ultrasound revealed small amounts of peritoneal fluid; uneven contours and diffusely abnormal structure of the liver, later interpreted as LC; and splenomegaly. With regard to WD as a potential differential diagnosis, a genetic consultation recommended the performance of genetic testing. Meanwhile, the patient received symptomatic and corticosteroid therapy for the suspected autoimmune hepatitis (AIH) and was discharged from the hospital 30 days after the admission with improvement of the general status. Three days later, the teen experienced deterioration with acute hemoptysis and pyrosis and was referred for immediate rehospitalization. The patient was readmitted to the hospital in a critical state. Reanimation measures had a temporary effect, and ultimately exitus letalis was registered. The results of the ATP7B gene sequencing, which were obtained a few days after the exitus, supported the clinical diagnosis and the autopsy suspicion, showing that the boy was homozygous to an H1069Q mutation. 

The autopsy study revealed the following morphological changes as a result of untreated WD in the child: mixed micronodular and macronodular LC, chronic steatohepatitis, hepatosplenomegaly, ascites, icterus, gynecomastia, telangiectasias, subcutaneous hemorrhages, absence of male pattern body hair, hypogonadism, and chronic calculous cholecystitis. 

Clinically, the condition was complicated by hepatorenal syndrome. Other complications of the main disease appeared to be severe bilateral purulent-hemorrhagic pneumonia, probably with mixed etiology, acute cardiac failure with congestive changes in all internal organs, pleural and pericardial effusions, pulmonary edema, and cerebral edema with tonsillar herniation. The latter was recognized as the immediate cause of death. 

Grossly, the liver with a weight of 1792 g was irregularly shaped, with numerous nodules of varying dimensions and chromaticity, from pale yellow to greenish to greenish-bluish. The thick-walled gallbladder was filled with green bile and contained dark green concrements. The spleen was dark red and enlarged in size (534 g). Both kidneys had a relatively smooth capsule and got easily decapsulated, dark reddish in color underneath. The pericardial sac was intact and contained approximately 100 ml of clear straw-yellow liquid. The heart was increased in size (305 g) and its internal texture was slightly accentuated. The myocardium, which was reddish-brown in section, demonstrated dense consistency. Bilateral pleural effusions with a volume of approximately 200 ml each were reported. On the surface of the cerebellar tonsils, a groove was distinguished, as a result from the cerebral edema and the following tonsillar herniation into foramen magnum. 

**Figure 1:**
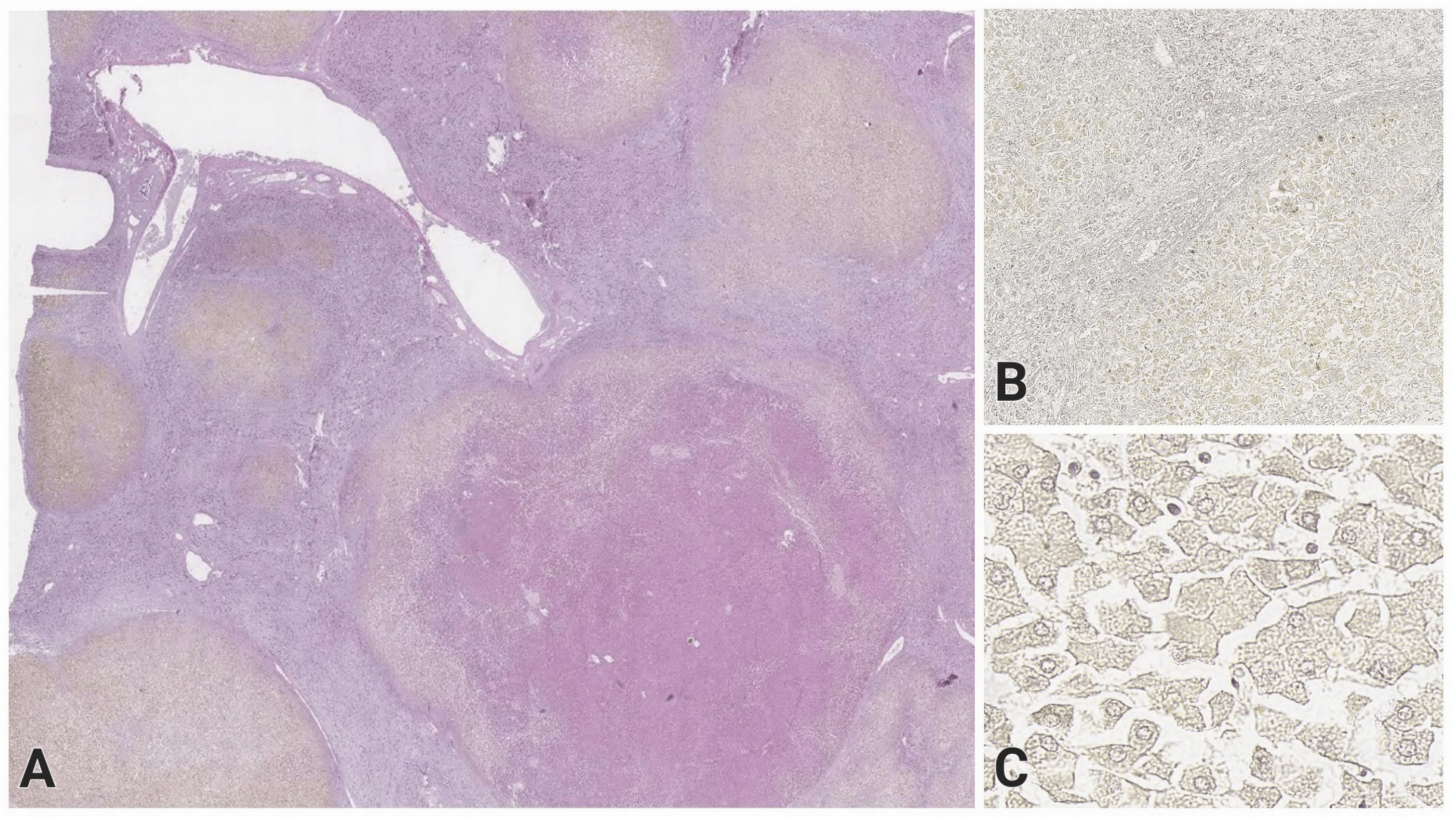
Images of the changes in the liver on histological examination made with a digital slide scanner and the pathology slide viewing software Aperio ImageScope H&E: Hematoxylin and eosin (A) Mixed liver cirrhosis, presenting with regenerative nodules of varying size, demarcated by thick fibrous septa, H&E, original magnification x10. (B) Positive Rhodamine staining for intracytoplasmic copper depositions in the hepatocytes, original magnification x30. (C) Higher magnification of the Rhodamine staining with positive histochemical reaction, original magnification x200

Microscopically, abundant regenerative nodules were found in the liver, demarcated by dense fibrous septa with bile duct proliferation and lymphoid aggregates (Figure 1A). Large nodules with deposition of Rhodamine-positive and Perls-negative cytoplasmic pigment in hepatocytes were observed (Figures 1B, 1C). Hepatocytes showed degenerative changes: increased size, multinucleation, and intracytoplasmic fat droplets. Intrahepatic cholestasis was prominent. Scattered Malory bodies were seen on the specimen.

**Figure 2:**
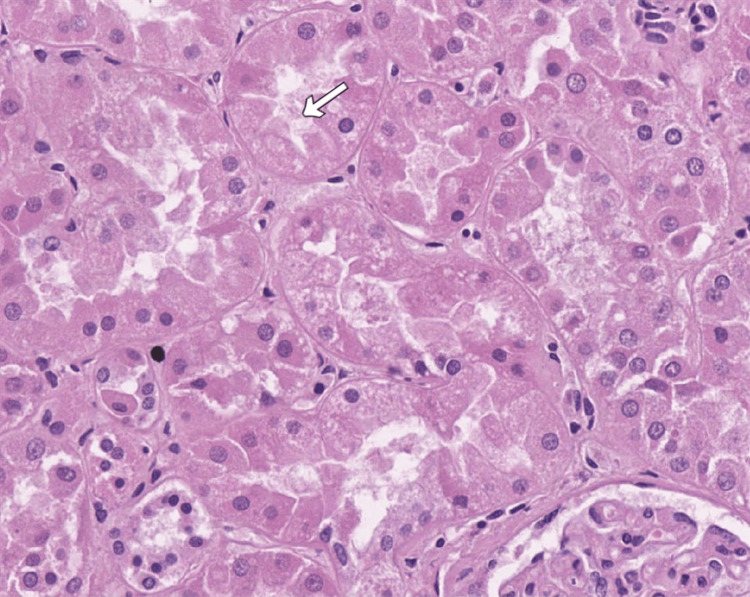
Scanned image of the histology of the kidneys with focal acute tubular necrosis, H&E staining, original magnification x150 H&E: Hematoxylin and eosin Focal acute tubular necrosis (white arrow) with the presence of desquamated epithelial cells in some of the tubular lumens

In the kidneys, there was flattening of the epithelial cells of the proximal renal tubules with dilation of the tubular lumens. Focal acute tubular necrosis with the presence of desquamated epithelial cells was observed in some of the tubular lumens (Figure 2).

**Figure 3:**
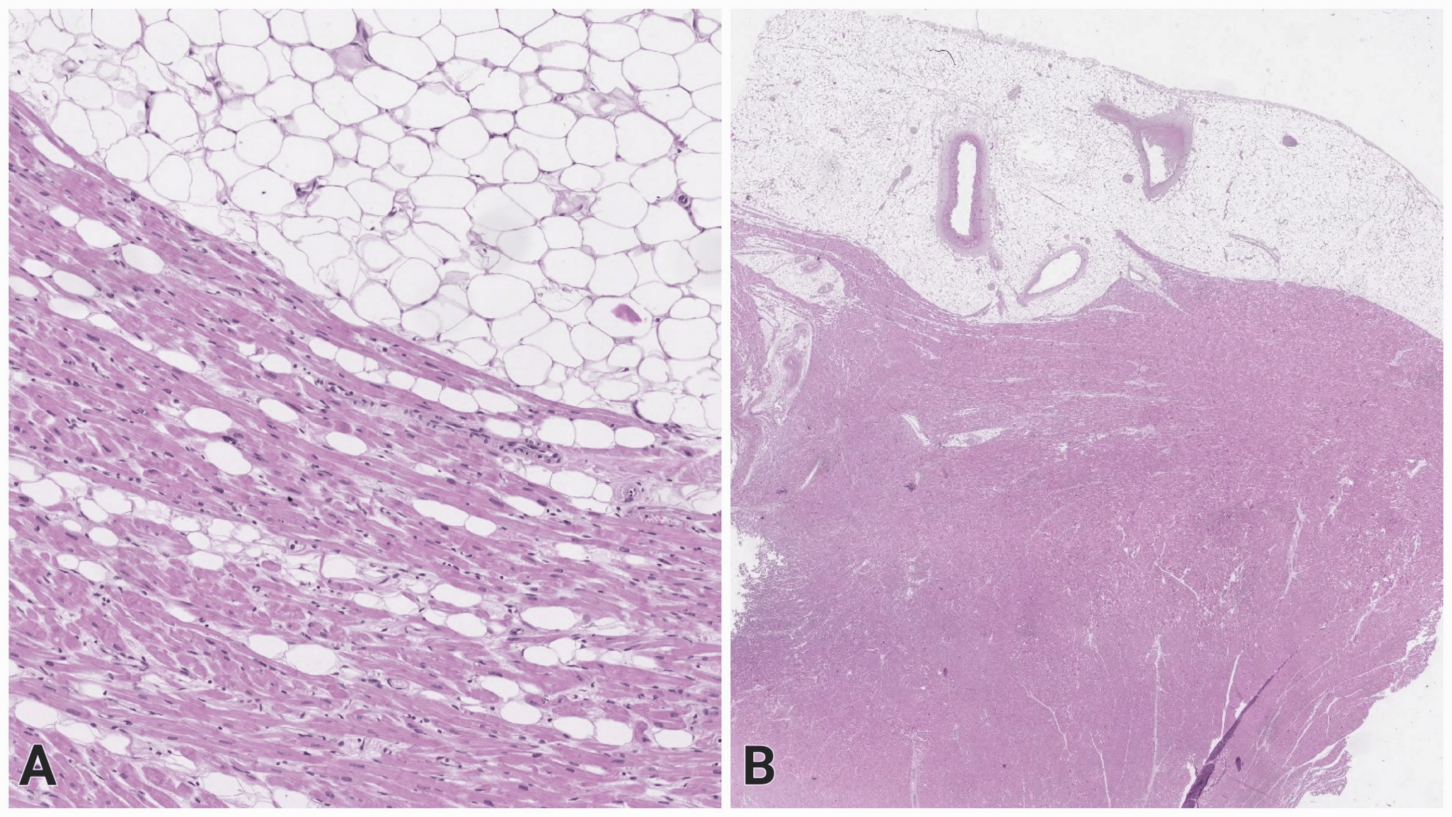
Scanned pathology slide, showing the histologic appearance of the heart, H&E H&E: Hematoxylin and eosin (A) Myocardium with hypertrophied cardiomyocytes, interstitial fibroblasts within collagenous septa, and subepicardial lipomatosis, original magnification x50. (B) Thick myocardial wall with excessive overlying epicardial fat, original magnification x10

The spleen presented with marked blood stasis, multiple hemorrhagic foci, and atrophic lymphoid follicles. In the thick myocardium, hypertrophied cardiomyocytes and interstitial fibroblasts within collagenous septa were seen (Figure 3A). Abundant adipocytes were present in the epicardial adipose tissue, which is an unusual finding in children (Figure 3B). 

**Figure 4:**
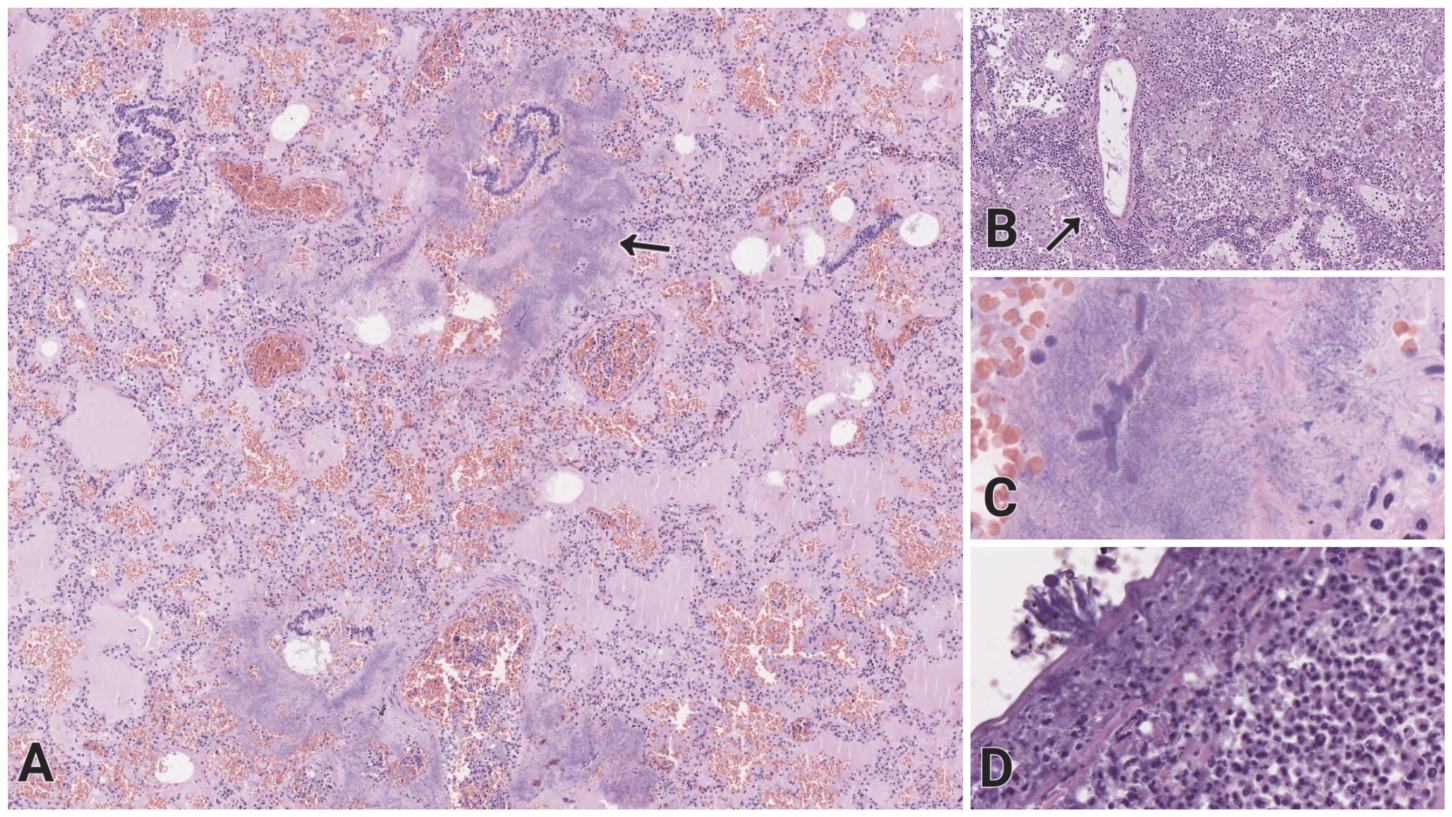
Histopathology of the lungs with purulent-hemorrhagic pneumonia of mixed etiology, captured on Aperio ImageScope slide viewing software, H&E staining H&E: Hematoxylin and eosin (A) Pulmonary parenchyma with pale pink fluid diffusely in the alveoli with optically empty air bubbles, numerous erythrocytes, alveolar macrophages, neutrophilic leukocytes, and abundant colonies (arrow), original magnification x50. (B) Lymphoplasmacytic cuffs around arterial vessels (arrow), original magnification x100. (C) Intraalveolar bacterial colonies and mycotic hyphae with hemorrhagic background, original magnification x400. (D) Visceral pleura with mycotic microorganisms and underlying mixed inflammatory infiltrate, original magnification x200

The lungs demonstrated hyperemic capillary vessels and pale pink fluid diffusely in the alveoli with optically empty air bubbles, numerous erythrocytes, alveolar macrophages, and neutrophilic leukocytes (Figure 4A). The histological examination also displayed single fibrin deposits, vessels with bone marrow emboli, lymphoplasmacytic cuffs around arterial walls (Figure 4B), hemorrhages, and extensive bacterial and mycotic colonies in vessels, among parenchyma and over the visceral pleura (Figures 4A, 4C, 4D).

**Figure 5:**
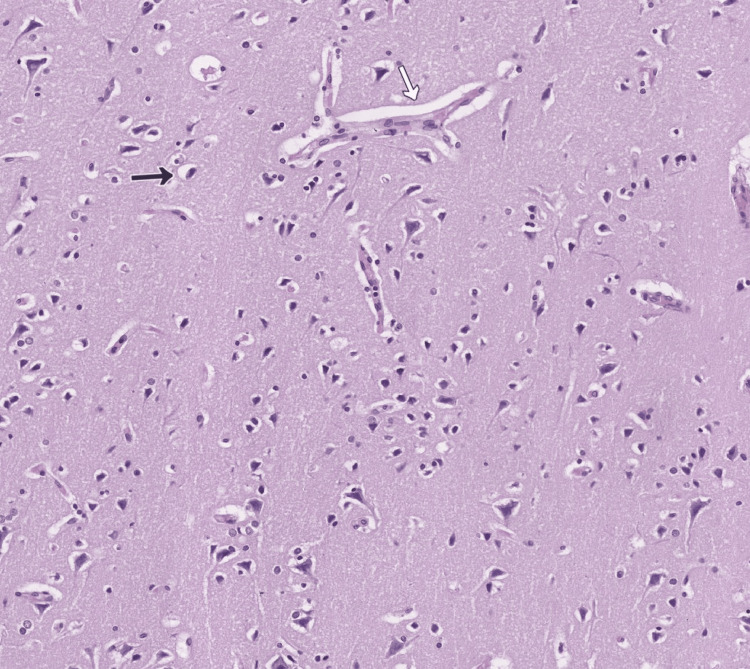
Brain tissue expressing signs of cerebral edema, H&E, original magnification x50 H&E: Hematoxylin and eosin Hyperemic vessels with perivascular (white arrow) and pericellular (black arrow) optically empty spaces, representing dyscirculatory changes in the cerebrum

The brain exhibited preserved architecture with dyscirculatory changes: hyperemia and pericellular and perivasal lucency due to the cerebral edema (Figure 5).

## Discussion

As previously mentioned, WD is an autosomal recessive genetic disease, which means that the proband has received one mutated copy of the ATP7B gene from each parent or, rarely, is a result of uniparental isodisomy with two dysfunctional gene copies. Generally, autosomal recessive disorders are more commonly observed in small ethnic groups due to reduced genetic diversity and a higher frequency of consanguinity, increasing the risk of homozygosity. In our case, the patient was a member of the Roma community, which, in addition to endogamy, is characterized by a high birth rate. A Bulgarian study from 2005 showed significant genetic homogeneity in 15 unrelated families of Romani people with WD, 100% of them having the same H1069Q mutation in the ATP7B gene [5]. 

Clinically, WD may create some diagnostic difficulties. In children with this disease, the Kayser-Fleischer rings are usually absent, as in the present case. Hemolytic anemia, as a result of the rapid release of free copper in the bloodstream from damaged hepatocytes and subsequent erythrocyte destruction, is possible and can be detected as high urobilinogen levels in the urine and typical anemic syndrome in blood tests. Moreover, during the diagnostic process, WD should not be excluded in the presence of infection, AIH, or nonalcoholic fatty liver disease [6]. Patients with WD may have elevated levels of antinuclear antibodies (ANA), anti‐neutrophil cytoplasmic antibodies (ANCA), anti‐muscle‐specific tyrosine kinase antibodies, and anti‐acetylcholine receptor antibodies, particularly those undergoing D‐penicillamine chelation therapy. These findings may be misleading, and potential immunosuppressive therapy with corticosteroids for AIH usually does not result in improvement but increases the risk of infections and other steroid-induced adverse effects [7,8]. In rare cases, coexistence between WD and AIH has been observed. The combined treatment of steroids and D-penicillamine has demonstrated efficacy in such instances [9]. Overlap syndromes with other conditions (e.g., sickle cell trait) leading to complex presentations of WD have also been described in the literature [10]. 

Furthermore, histological examination has certain limitations in terms of confirming the diagnosis. Specific histochemical stains for copper, such as Timms sulfur, Rhodamine, and Orcein, with the former being the most sensitive, often fail to detect copper deposition on liver biopsies because they estimate lysosomal copper stores only [11]. A more reliable indicator of hepatic copper overload is the hepatic copper concentration with a cutoff value of 250 μg/g dry weight [12]. This biochemical method is believed to be useful, but values below 250 do not exclude WD [13].

## Conclusions

In conclusion, WD is a relatively rare genetic disease with a broad spectrum of clinical manifestations, especially in children and cases of overlapping conditions. This makes WD hardly recognizable and challenging for treatment in some situations, particularly in ethnic groups with numerous risk factors, low educational status, financial hardship, and a lack of prevention measures. Consequently, the prognosis of this potentially treatable disease may become unfavorable, which increases the mortality at a younger age.
